# Telomere Length in Peripheral Blood Leukocytes Is Associated with Severity of Biliary Atresia

**DOI:** 10.1371/journal.pone.0134689

**Published:** 2015-07-31

**Authors:** Wanvisa Udomsinprasert, Yong Poovorawan, Voranush Chongsrisawat, Paisarn Vejchapipat, Dong Zhan, Sittisak Honsawek

**Affiliations:** 1 Department of Biochemistry, Faculty of Medicine, Chulalongkorn University, King Chulalongkorn Memorial Hospital, Thai Red Cross Society, Bangkok 10330, Thailand; 2 Center of Excellence in Clinical Virology, Department of Pediatrics, Faculty of Medicine, Chulalongkorn University, King Chulalongkorn Memorial Hospital, Thai Red Cross Society, Bangkok 10330, Thailand; 3 Department of Surgery, Faculty of Medicine, Chulalongkorn University, King Chulalongkorn Memorial Hospital, Thai Red Cross Society, Bangkok 10330, Thailand; University of Newcastle, UNITED KINGDOM

## Abstract

**Objective:**

The purpose of this study was to investigate the association of telomere length in peripheral blood leukocytes with the severity of biliary atresia (BA).

**Methods:**

One hundred and fourteen BA patients and 114 age-matched healthy controls were enrolled. Relative telomere length (RTL) was assessed using a quantitative real-time polymerase chain reaction. Multivariate regression analysis was used to estimate RTL as an independent risk factor of BA. Receiver operating characteristic curve analysis was used to calculate the accuracy of biomarkers in the prediction of liver cirrhosis.

**Results:**

BA patients had significantly shorter telomeres than healthy controls (*p* < 0.0001). The RTL in BA patients with jaundice was considerably lower than that of patients without jaundice (*p* = 0.005). Moreover, RTL was markedly shorter in patients with cirrhosis (F4), as compared to patients with mild fibrosis (F2) and non-fibrosis (F0-F1, *p* < 0.0001). Logistic regression analysis indicated that short RTL was associated with a higher risk of liver cirrhosis in BA. Tertile analysis showed a dose-response effect for this association (*p* trend < 0.0001). Additionally, RTL in BA children revealed a negative correlation with age (*r* = -0.50, *p* < 0.001). We noted an association between reduction of RTL and liver stiffness scores, adjusted for age and gender (*b* = -0.01, *p* < 0.0001). Short RTL can be employed to distinguish cirrhosis patients from non-cirrhosis patients (AUC = 0.78). Further analysis showed a linear correlation between leukocyte RTL and liver RTL in BA patients (*r* = 0.83, *p* < 0.001).

**Conclusion:**

The findings of this study provide evidence that telomere shortening is associated with an elevated risk of liver cirrhosis in BA.

## Introduction

Biliary atresia (BA), the most common cause of cholestatic liver disorder in infants, is characterized by progressive fibrosclerosing cholangiopathy affecting the extra- and intrahepatic biliary ducts. BA patients who experience obstruction of bile flow suffer persistent jaundice, acholic stools, hepatomegaly, and/or splenomegaly. If left untreated, the majority of BA children will develop chronic liver disease (severe hepatic fibrosis, biliary cirrhosis, and liver failure) and most likely die by the age of 2 years [1]. Kasai portoenterostomy, the first-line intervention for infants with BA, reestablishes bile flow to the gastrointestinal tract. Liver transplantation is another treatment option in cases where Kasai portoenterostomy fails or is not practical [2]. The precise etiology and pathophysiology of BA remains elusive. Environmental factors may be a cause of BA in a genetically susceptible individual during early infancy. If this is the case, variants of genes playing a role in hepatobiliary development or immunological tolerance tend to be candidates for mediating susceptibility. Moreover, evidence supporting the role of genetic factors as a cause of BA has been accumulating for a number of years [3, 4]. In addition to results from epidemiological studies, polymorphism studies, and data on twins, the concept of shortened telomere length as a genetic risk factor for liver fibrosis and BA has been proposed.

Telomeres, which are located at the ends of chromosomes, consist of repetitive DNA sequences of TTAGGG and related proteins of crucial importance for telomere function. Telomeres help maintain genomic integrity and stability by shielding chromosome ends from deterioration, fusion, and atypical recombination [5]. The telomere length shortens each time cells divide, because DNA polymerases are not capable of completely replicating chromosomes during cell division. This is commonly referred to as the end-replication problem. This alteration in telomere length precipitates capping function losses at the chromosomal ends, leading to DNA damage program activation, which contributes to senescence, apoptosis, and neoplastic transformation [6]. As such, telomere length is an indicator of the biological age of a cell.

There is also emerging evidence that describes an association between attrition of telomere length and several human pathologies [7, 8], including a variety of cancers and chronic liver disorders, such as liver hepatitis, cirrhosis, and hepatocellular carcinoma (HCC) [9–11]. These findings strongly suggest telomere shortening in the development of liver cirrhosis. Accordingly, evaluation of telomere length may serve as a feasible and reliable non-invasive indicator for determining the risk and prognosis of BA. In support of this proposed causal relationship, a previous study demonstrated telomere shortening in liver tissues of BA patients at the time of liver transplantation [12]. Until now, no report has specifically examined the relationship between telomere length in peripheral blood leukocytes and biochemical parameters in BA patients, particularly by considering DNA from leukocytes as a non-invasive biomarker. This proposed method would provide a cost-effective and time-saving alternative, as peripheral blood leukocytes are easier to collect and evaluate than liver tissue.

In this study, quantitative real-time polymerase chain reaction (PCR) was used to compare and evaluate telomere length in patients with BA and age-matched healthy controls. We hypothesized that shortened telomere length can be positively correlated with increased severity of BA. To prove this hypothesis, we investigated telomere length in peripheral blood leukocytes from both BA patients and age-matched healthy controls and evaluated the association between telomere length and clinical parameters of BA patients.

## Materials and Methods

### Study population

This cross-sectional analytical study was composed of 114 patients with BA (66 females and 48 males) and 114 healthy age-matched controls (64 females and 50 males). BA patients who came for the follow-up visit to the Pediatric Liver Clinic were enrolled. All BA patients were diagnosed using intra-operative cholangiography and were surgically treated with original Kasai portoenterostomy. BA children who had undergone liver transplantation were excluded from this study. Age-matched unaffected volunteers who had normal physical findings and no underlying disease were included as the controls. In addition, two pairs of monozygotic twins with BA discordance (one set of whom suffered from BA) were recruited for this investigation. The BA children were stratified in terms of bile flow establishment into a non-jaundice group (TB < 2 mg/dl) and a persistent jaundice group (TB ≥ 2 mg/dl), according to serum total bilirubin (TB). Based on the severity of liver fibrosis (liver stiffness values), BA patients were also divided into four groups: non-fibrosis (F0-F1: 0–7.2 kPa), mild fibrosis (F2: 7.3–9.7 kPa), severe fibrosis (F3: 9.8–17.2 kPa), and cirrhosis (F4: ≥ 17.3 kPa), as previously described by Corpechot et al. [13].

The liver function tests including serum albumin, total bilirubin (TB), aspartate aminotransferase (AST), alanine aminotransferase (ALT), and alkaline phosphatase (ALP) were performed by a Hitachi 912 Chemistry Analyzer at the central laboratory of our hospital. Blood samples from every participant were drawn using ethylenediaminetetraacetic acid for anticoagulation. After centrifugation at 4,000 rpm for 10 minutes, the blood was separated into plasma and leukocytes. The plasma and leukocytes were stored at -80(C until further analysis. Due to the availability to collect liver tissue of BA patients in some cases, we obtained only 6 liver tissue samples and matched DNA samples from peripheral blood leukocytes of BA patients. All liver tissue samples were immediately frozen and stored at -80°C for further measurement.

The protocol for this study was approved by the Institutional Review Board of the Faculty of Medicine, Chulalongkorn University (IRB number 279/57). This study was conducted in accordance with the ethical standards outlined in the 1975 Declaration of Helsinki. All participants, parents, or legal guardians were fully informed regarding the study protocol and procedures prior to participating in the study. Written informed consent was obtained from the participants’ parents upon informing them about the protocol and procedures involved in the research.

### Measurement of telomere length

Telomere length in genomic DNA extracted directly from peripheral blood leukocytes and liver tissue according to the instruction of DNA extraction kit (GE Healthcare, Buckinghamshire, UK) was measured by applying a quantitative real-time PCR method, as previously described by Cawthon et al. [14]. Telomere length was measured according to the ratio of the telomere repeat copy number (T) to the single-copy gene copy number (S) in each given sample. The single-copy gene refers to the 36B4 gene, which encodes the acid ribosomal phosphoprotein (PO). The ratio (T/S) is proportional to the average telomere length. DNA samples were amplified in 10 (l PCR reactions with StepOnePlus Real Time PCR system (Applied Biosystems, Foster City, CA, USA). The primers used for the telomere repeat copy number and the single-copy gene copy number amplification were, as follows: telomere forward 5′-CGGTTTGTTTGGGTTTGGGTTTGGGTTTGGGTTTGGGTT-3′; telomere reverse 5′-GGCTTGCCTTACCCTTACCCTTACCCTTACCCTTACCCT-3′; single-copy gene forward 5′-CAGCAAGTGGGAAGGTGTAATCC-3′; and, single-copy gene reverse 5′-CCCATTCTATCATCAACGGGTACAA-3′. Both PCRs were activated in a final volume of 10 (l that contained SYBRGreen Master Mix none-ROX (2x) (RBC Bioscience, Taipei, Taiwan), 3.12 ng of DNA template, and 0.5 nM of telomere primers or 0.5 nM of single-copy gene primers. The thermal cycling profile for both telomeres and single copy genes started with 95°C incubation for 10 min, followed by 40 cycles of 15 sec at 95°C and 1 min at 54°C. All amplification specificity was regulated by employing melting curve analysis. In each sample, the quantity of telomere repeats and the quantity of single-copy genes were normalized to a reference DNA. The same reference DNA sample (from a single individual) was included in each measurement to control inter-assay variability.

### Liver stiffness assessment

The assessment of liver stiffness was performed on the same day as blood sampling. Transient elastography determined the liver stiffness between 25 and 65 mm from the skin surface. The measurements were performed by placing a transducer probe of Fibroscan (EchoSens, Paris, France) on the intercostal space at the area of the right lobe of the liver. Measurements were then performed until 10 validated results were obtained with a success rate of at least 80%. The median value of 10 validated scores represented the elastic modulus measurement of the liver and it was expressed in kilopascals (kPa) [15].

### Statistical analysis

Statistical analyses were performed with the SPSS statistical package, version 20.0 (SPSS Inc., Chicago, IL, USA). The Kolmogorov-Smirnov test and quantile-quantile plot were used to assess whether relative telomere length (RTL) was normally distributed. Comparisons between means were evaluated by Student’s *t*-test, while the Mann-Whitney *U* test and Kruskal-Wallis H test were employed for comparison of abnormally distributed continuous variables. Spearman’s rank correlation coefficient test was used to define the relationship between telomere length and age. The associations of RTL with the risk of BA were measured by applying univariate and multivariate logistic regression analyses to determine the roles of confounding factors. Receiver operating characteristic (ROC) curves were constructed to evaluate the specificity and sensitivity of predicting cirrhosis using RTL values, and the area under curve (AUC) was calculated. Data are presented as mean ± standard error of the mean. For all statistics, a *p*-value less than 0.05 (based on a two-tailed test) was considered statistically significant.

## Results

### Characteristics of the study participants

The baseline characteristics of the 114 BA patients and 114 unaffected volunteers are summarized in Table 1. Participants were age-matched between BA patients and healthy controls. Although the number of females was higher than males in both controls and BA patients, there was no significant difference. As expected, liver stiffness values in BA patients were substantially higher than those in controls (*p* < 0.0001). In addition, there were significantly higher serum AST and ALT levels in BA patients than in controls (*p* < 0.0001).

**Table 1:** Clinicopathologic characteristics of biliary atresia patients and age-matched healthy controls.

	BA patients (n = 114)	Controls (n = 114)	*p*-value
Age (years)	8.95 (0.45	8.95 (0.45	NS
Gender (female:male)	66:48	64:50	NS
Albumin (g/dl)	4.04 (0.09	-	NA
Total bilirubin (mg/dl)	2.72 (0.37	-	NA
AST (IU/l)	117.92 (9.27	26.66 (0.82	< 0.0001
ALT (IU/l)	97.23 (8.38	9.24 (0.65	< 0.0001
ALP (IU/l)	421.00 (29.74	-	NA
Liver stiffness (kPa)	32.78 (2.38	4.01 (0.19	< 0.0001

Abbreviations: BA = biliary atresia; AST = aspartate aminotransferase; ALT = alanine aminotransferase; ALP = alkaline phosphatase; NS = not significant; NA = not available

### Relative telomere length distribution in the study subjects

**Fig 1:**
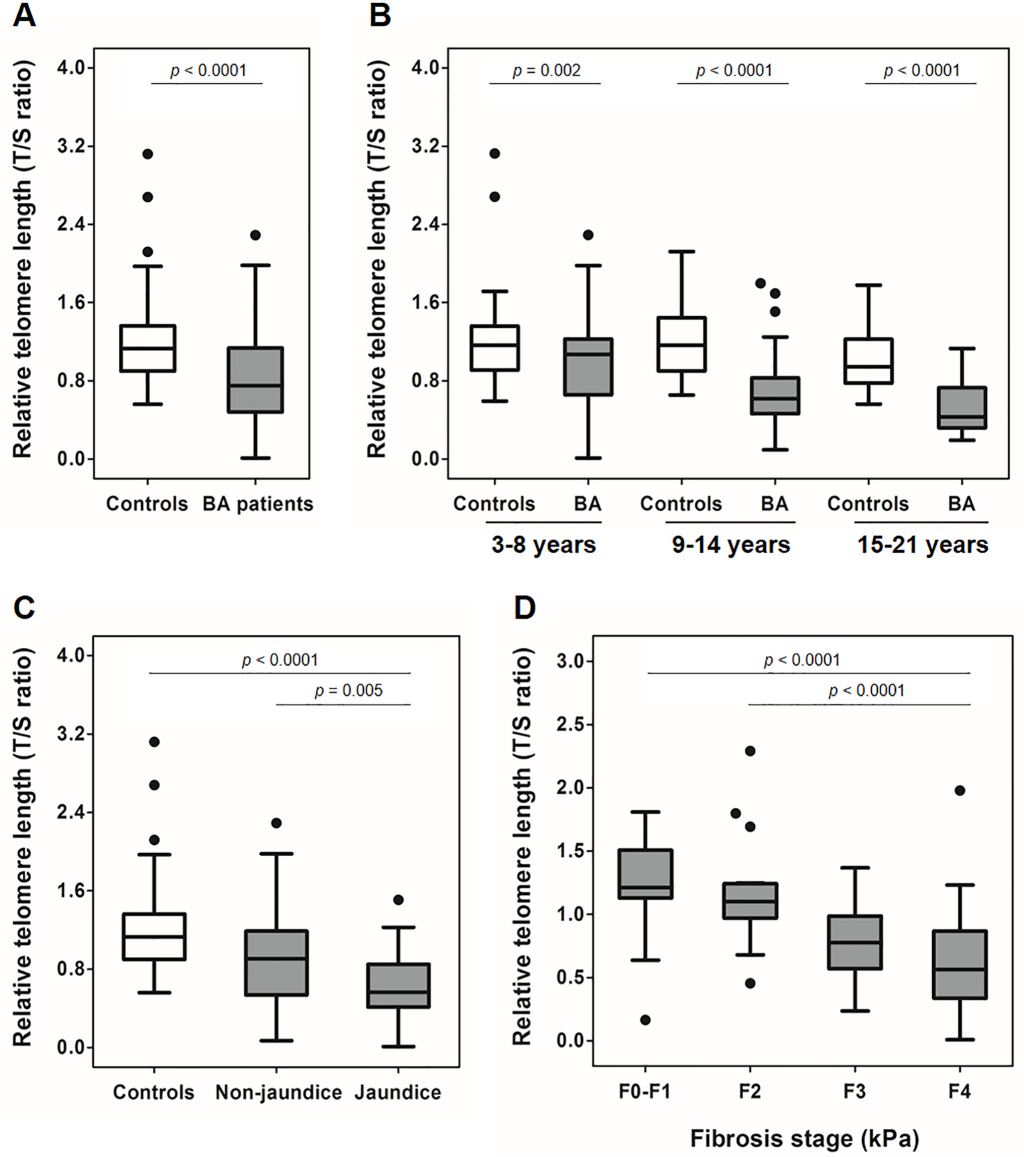
Box-plot illustrating telomere length distribution in subjects among different groups: The line through the middle of the boxes represents the median of T/S value and the top and bottom of each box represents the first and third quartiles. The lower and upper error bars are computed as the lower and upper quartiles, respectively. (A) Relative telomere length in BA patients and healthy controls; (B) Relative telomere length in BA patients and controls, according to age group; (C) Relative telomere length in patients with and without jaundice; (D) Relative telomere length in BA subgroups, including non-fibrosis (F0-F1), mild fibrosis (F2), severe fibrosis (F3), and liver cirrhosis (F4).

We investigated telomere length in leukocytes from the BA group and the unaffected controls. Overall, the RTL in leukocytes was significantly lower in BA children compared to healthy controls (*p* < 0.0001), as shown in Fig 1A. Given that telomere length is age-related, we classified the subjects of both groups into 3 age categories (3 to 8 years, n = 60; 9 to 14 years, n = 40; and, 15 to 21 years, n = 14). A significantly shortened telomere length could be found in BA patients within each of the 3 age categories, as compared to the control group (*p* = 0.020, *p* < 0.0001, and *p* < 0.0001, respectively), as presented in Fig 1B.

In stratified analysis according to jaundice status, BA patients were divided into persistent jaundice and non-jaundice groups (Table 2). Interestingly, the RTL in BA patients with persistent jaundice was markedly shorter than that in BA patients without jaundice (*p* = 0.005). Furthermore, there was a significant difference in RTL between BA patients with jaundice and healthy controls (*p* < 0.0001). We also found that BA patients without jaundice had markedly shorter telomere length than unaffected volunteers (*p* < 0.0001) (Fig 1C).

**Table 2:** Clinicopathologic characteristics of biliary atresia patients with and without jaundice.

	BA patients (n = 114)		*p*-value
	Non-jaundice (n = 77)	Jaundice (n = 37)	
Age (years)	8.38 ± 4.32	9.31 ± 0.94	NS
Gender (female:male)	46:31	20:17	NS
Albumin (g/dl)	4.14 ± 0.11	3.78 ± 0.12	0.032
Total bilirubin (mg/dl)	0.77 ± 0.13	7.05 ± 0.74	< 0.0001
AST (IU/l)	90.87 ± 19.24	186.13 ± 19.24	< 0.0001
ALT (IU/l)	94.76 ± 10.04	131.97 ± 12.59	0.024
ALP (IU/l)	358.64 ± 31.62	586.68 ± 58.17	0.001
Liver stiffness (kPa)	26.93 ± 2.78	45.44 ± 3.83	< 0.0001

Abbreviations: BA = biliary atresia; AST = aspartate aminotransferase; ALT = alanine aminotransferase; ALP = alkaline phosphatase; NS = not significant

We further explored telomere length in leukocytes from a subgroup of BA patients according to liver stiffness value (F0-F1: 0–7.2 kPa, n = 15; F2: 7.3–9.7 kPa, n = 17; F3: 9.8–17.2 kPa, n = 18; and, F4: ≥ 17.3 kPa, n = 69). Table 3 illustrates the clinical characteristics of the BA subgroups based on the severity of liver fibrosis. BA children with cirrhosis (F4) had significantly greater telomere shortening than both patients with mild fibrosis (F2, *p* < 0.0001) and patients without liver fibrosis (F0-F1, *p* < 0.0001). However, RTL did not differ among BA children with severe fibrosis (F3) and other stages (Fig 1D).

**Table 3:** Clinicopathologic characteristics of biliary atresia patients with non-fibrosis, mild fibrosis, severe fibrosis, and liver cirrhosis.

	BA patients (n = 114)				*p*-value
	F0—F1 (n = 15)	F2 (n = 17)	F3 (n = 18)	F4 (n = 64)	
Age (years)	7.33 ± 0.74	8.52 ± 0.88	8.17 ± 1.13	9.62 ± 0.67	NS
Gender (female:male)	12:3	6:11	10:8	38:26	NS
Albumin (g/dl)	3.77 ± 0.35	3.90 ± 0.29	4.16 ± 0.24	4.13 ± 0.078	NS
Total bilirubin (mg/dl)	1.39 ± 0.86	1.34 ± 0.65	1.46 ± 0.50	3.77 ± 0.55	0.015
AST (IU/l)	68.53 ± 16.75	78.76 ± 16.91	100.94 ± 16.93	147.67 ± 13.97	0.0020
ALT (IU/l)	59.26 ± 14.38	74.52 ± 15.03	91.06 ± 13.67	128.46 ± 12.50	0.0060
ALP (IU/l)	300.26 ± 42.75	292.47 ± 53.97	386.23 ± 77.22	501.49 ± 43.92	0.010
Liver stiffness (kPa)	5.56 ± 0.19	8.75 ± 0.17	14.17 ± 0.54	50.57 ± 2.53	< 0.0001

Abbreviations: BA = biliary atresia; AST = aspartate aminotransferase; ALT = alanine aminotransferase; ALP = alkaline phosphatase; NS = not significant

### Relative telomere length in twins discordant for biliary atresia

**Fig 2:**
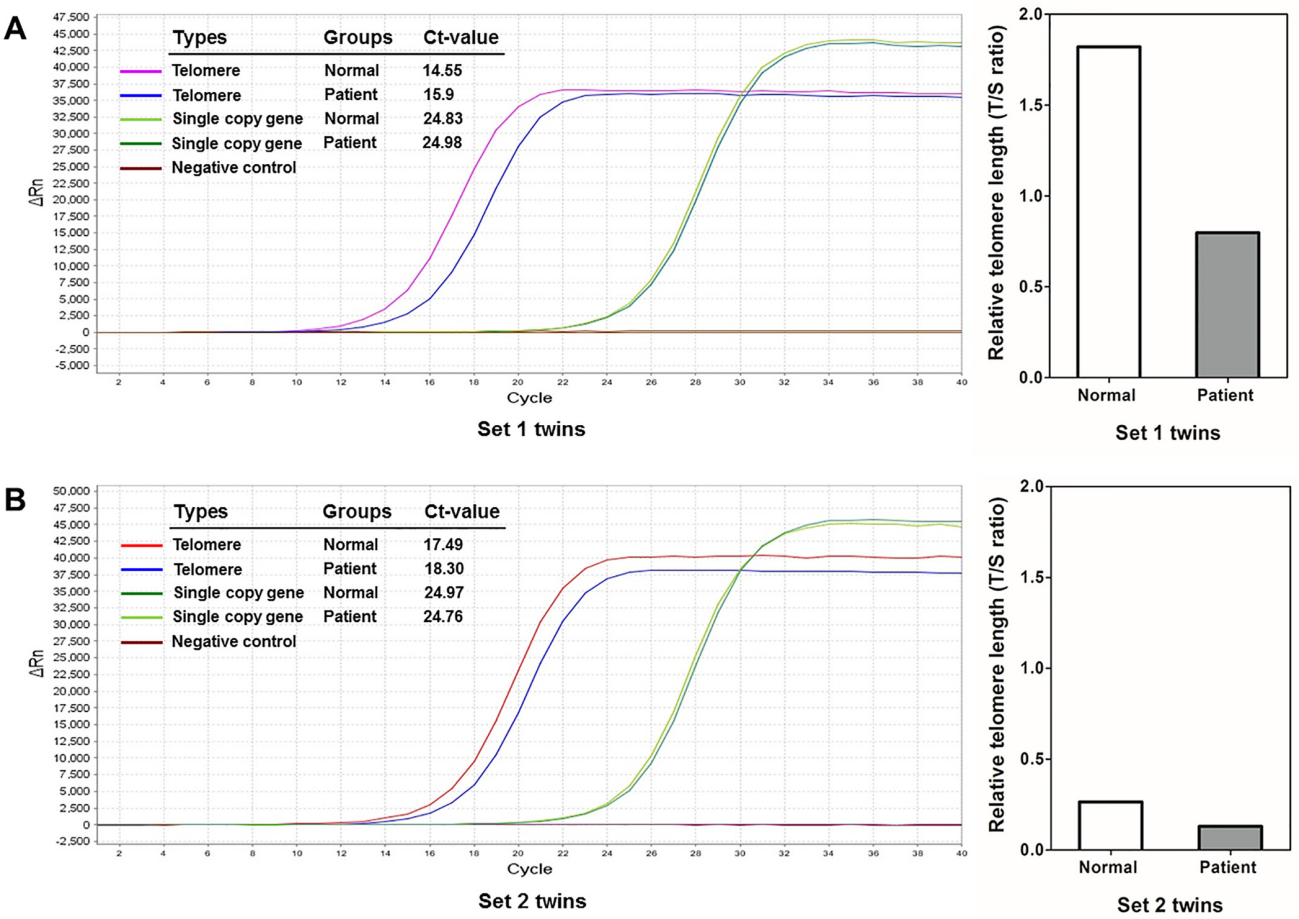
Telomere length assessment finding in two sets of twins by BA discordance: (A) Amplification plot and relative telomere length analysis in nine-year-old twin girls who were discordant for BA (set 1); (B) Amplification plot and relative telomere length analysis in nineteen-year-old twin women affected by BA discordance (set 2).

Subsequently, we examined telomere length in two sets of twins with discordant pathology in BA. Set 1: the patient is a nine-year-old girl who was diagnosed to have BA, with her twin sister being born healthy and remaining so to date. The RTL was found to be shorter in the BA twin, as compared to her healthy sister that served as the control group (T/S ratio: 0.80 vs. 1.82, respectively). Set 2: a case of 19-year-old twin women, one of whom suffers from BA. Her twin sister has remained healthy with normal liver function tests. We also observed that the BA twin had a shorter telomere length than her twin sister without BA (T/S ratio: 0.13 vs. 0.26, respectively), as demonstrated in Fig 2.

### Short relative telomere length and increased risk of BA

Since telomere length is also influenced by age and gender, we employed logistic regression analysis to control the role of confounding variables. After adjusting for age and gender, RTL in childhood BA was substantially shorter than that of the controls by an average of 0.089 units (95% CI: 0.038 to 0.21, *p* < 0.0001). The RTL of participants were separated into short RTL and long RTL groups, based on the median distribution of RTL in healthy controls. As shown in Table 4, patients with short RTL had a significantly elevated risk of BA, as compared to patients with long RTL in both univariate (unadjusted OR: 3.07, 95% CI: 1.75 to 5.39, *p* < 0.0001) and multivariate analysis (adjusted OR: 3.25, 95% CI: 1.82 to 5.81, *p* < 0.0001). We further categorized study subjects into three groups according to the tertile of RTL values in controls and investigated a significant dose-response association between short RTL and higher risk of BA. Specifically, using the third tertile (longest) as the reference group, the odds ratios (OR) for the first and second tertiles were 5.68 (95% CI: 2.65 to 12.16, *p* < 0.001) and 2.53 (95% CI: 1.14 to 5.64, *p* = 0.023), respectively, in unadjusted univariate analysis and 6.15 (95% CI: 2.82 to 13.42, *p* < 0.0001) and 2.54 (95% CI: 1.14 to 5.67, *p* = 0.023), respectively, in multivariate analysis. The *p* trend was less than 0.0001 in both analyses, suggesting quite strong evidence for a dose-response effect of short RTL-related higher risk of BA.

**Table 4:** Logistic regression analysis of association between relative telomere length and risk of biliary atresia.

RTL	BA	Controls	Unadjusted	*p*-value	Adjusted^a^	*p*-value
			OR (95% CI)		OR (95% CI)	
Overall	114	114	0.11 (0.048–0.24)	< 0.0001	0.089 (0.038–0.21)	< 0.0001
By median						
Short	86	57	3.07 (1.75–5.39)	< 0.0001	3.25 (1.82–5.81)	< 0.0001
Long	28	57	1 (reference)		1 (reference)	
By tertile						
1^st^ tertile	70	38	5.68 (2.65–12.16)	< 0.0001	6.15 (2.82–13.42)	< 0.0001
2^nd^ tertile	33	38	2.53 (1.14–5.64)	0.023	2.54 (1.14–5.67)	0.023
3^rd^ tertile	11	38	1 (reference)		1 (reference)	
*p* trend				< 0.0001		< 0.0001

Abbreviations: BA = biliary atresia; RTL = relative telomere length

^a^ Adjusted for age and gender

### Association between telomere length and clinical characteristics

**Fig 3:**
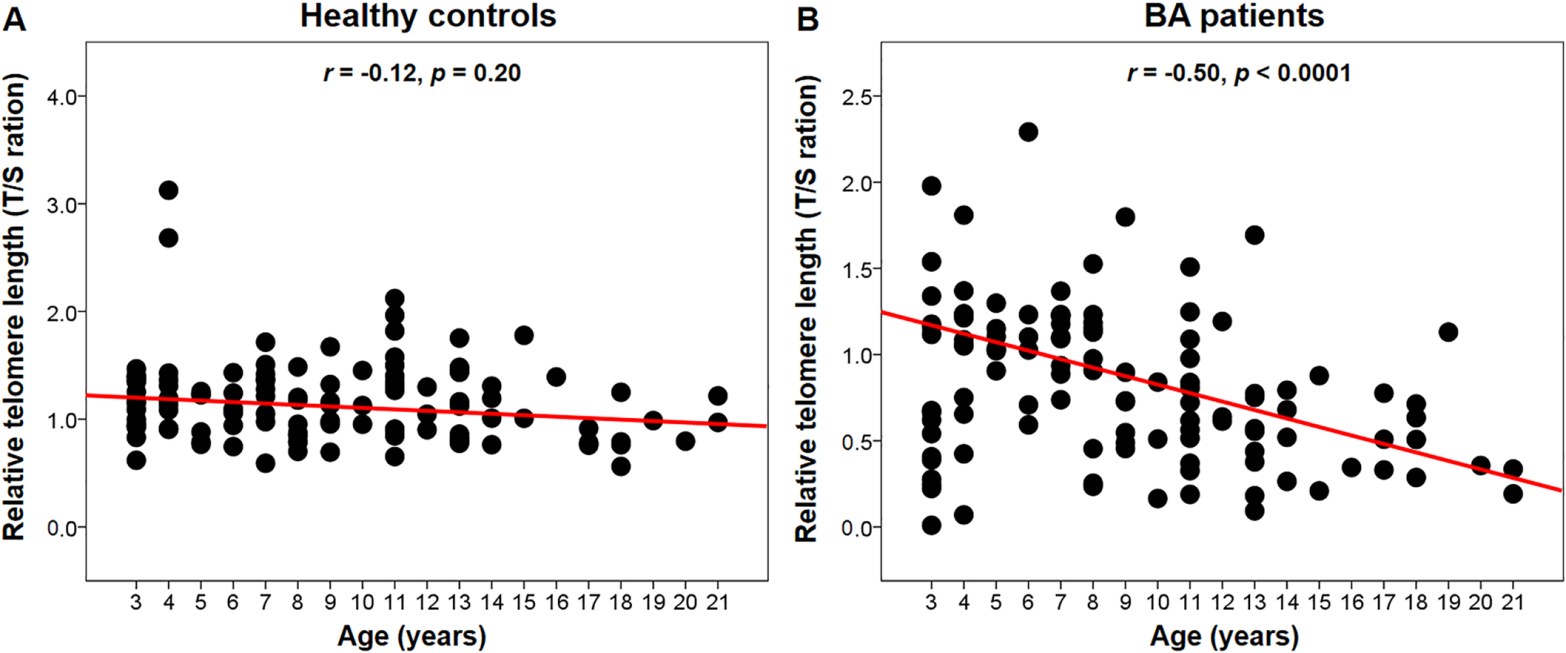
Scatter plot demonstrating correlation between relative telomere length of peripheral blood leukocytes and age in controls and BA patients: (A) Relative telomere length decrease with age in the controls; (B) Significant relative telomere length decrease with age in BA patients.

The association between RTL and age in BA patients and healthy controls is shown in Fig 3. As expected, no association between age and RTL was observed in healthy controls (*r* = -0.12, *p* = 0.20), while the RTL in BA patients showed an inverse association with age. There was a significant relationship between RTL and age, with RTL being longer in younger patients (*r* = -0.50, *p* < 0.001). We then performed multiple linear regression analysis, adjusting for age and gender to estimate the interaction between RTL and biochemical variables (Table 5). Interestingly, liver stiffness was found to be associated with a reduction in relative telomere length after adjusting for age and gender (*b* = -0.01, *p* < 0.0001).

**Table 5:** Multiple linear regression analysis of telomere length estimates.

Variables	Relative telomere length	*p*-value
	Estimate b (95% CI)	
Age (years)	-0.023 (-0.039 to -0.007)	0.005
Gender	0.028 (-0.11 to 0.17)	NS
Total bilirubin (mg/dl)	0.001 (-0.024 to 0.025)	NS
AST (IU/l)	0.00038 (-0.002 to 0.001)	NS
ALT (IU/l)	0.001 (-0.001 to 0.002)	NS
ALP (IU/l)	0.000076 (-0.00025 to 0.0004)	NS
Liver stiffness (kPa)	-0.01 (-0.013 to -0.007)	< 0.0001

Abbreviations: AST = aspartate aminotransferase; ALT = alanine aminotransferase; ALP = alkaline phosphatase; NS = not significant

### Shorter telomere length as prognostic marker for liver cirrhosis

**Fig 4:**
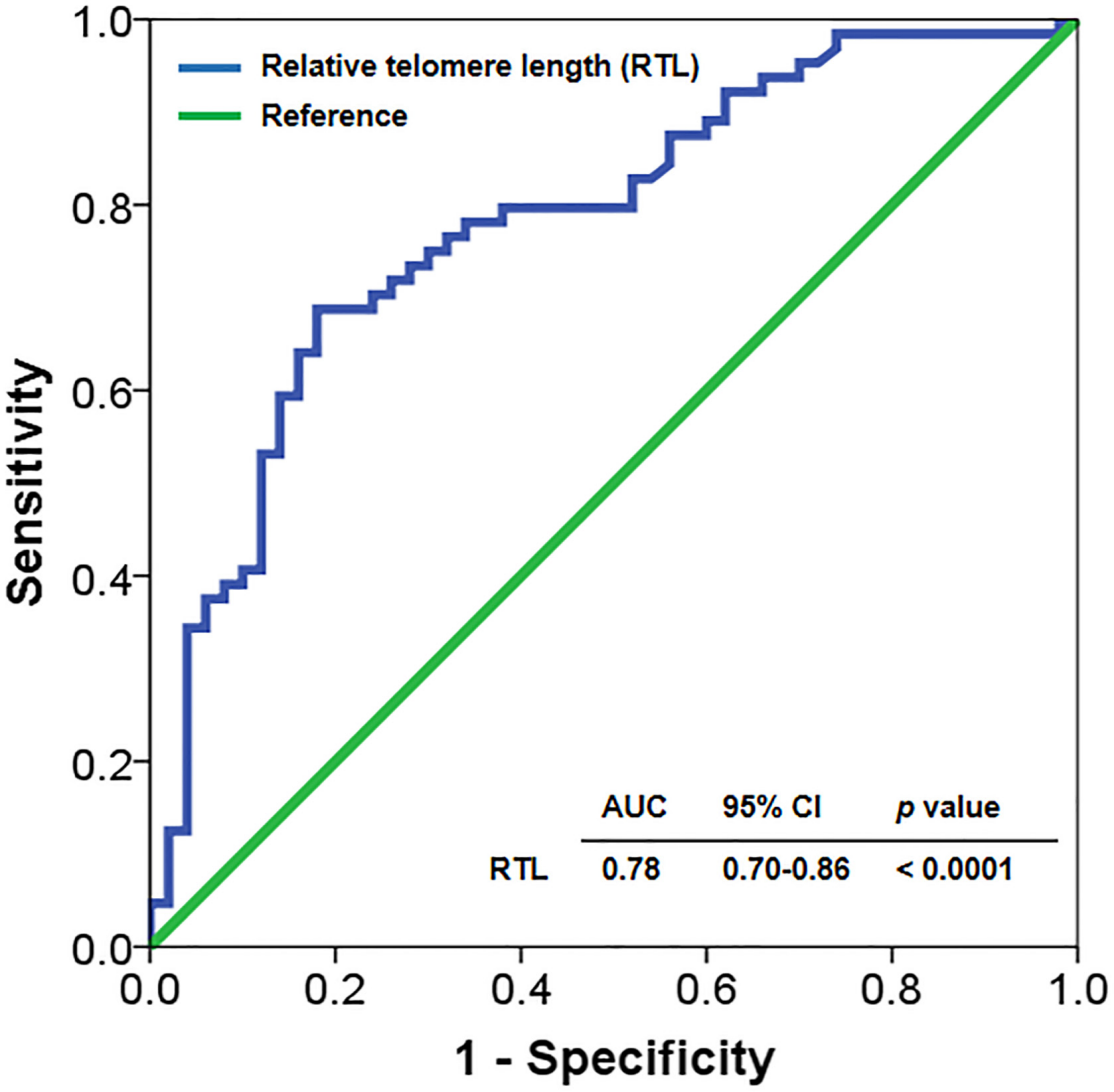
Receiver operating characteristic (ROC) curve representing diagnostic value of relative telomere length in biliary atresia patients with cirrhosis: The optimal cut-off value of relative telomere length at 0.58 as a marker discriminating between BA patients with and without cirrhosis.

Since RTL is an independent prognostic indicator, we further investigated RTL as a predictor of the risk of liver cirrhosis in postoperative BA patients. We calculated the area under curve (AUC) of the ROC curve, which was constructed using RTL values. Based on the ROC curve, the optimal cutoff value of RTL as a useful marker for discriminating BA patients with cirrhosis from non-cirrhosis BA patients was projected to be 0.58, which yielded a sensitivity of 76.6%, a specificity of 72%, and an AUC of 0.78 (95% CI: 0.70 to 0.86, *p* < 0.0001) (Fig 4).

### Correlation between telomere length in peripheral blood leukocytes and liver tissue in BA

**Fig 5:**
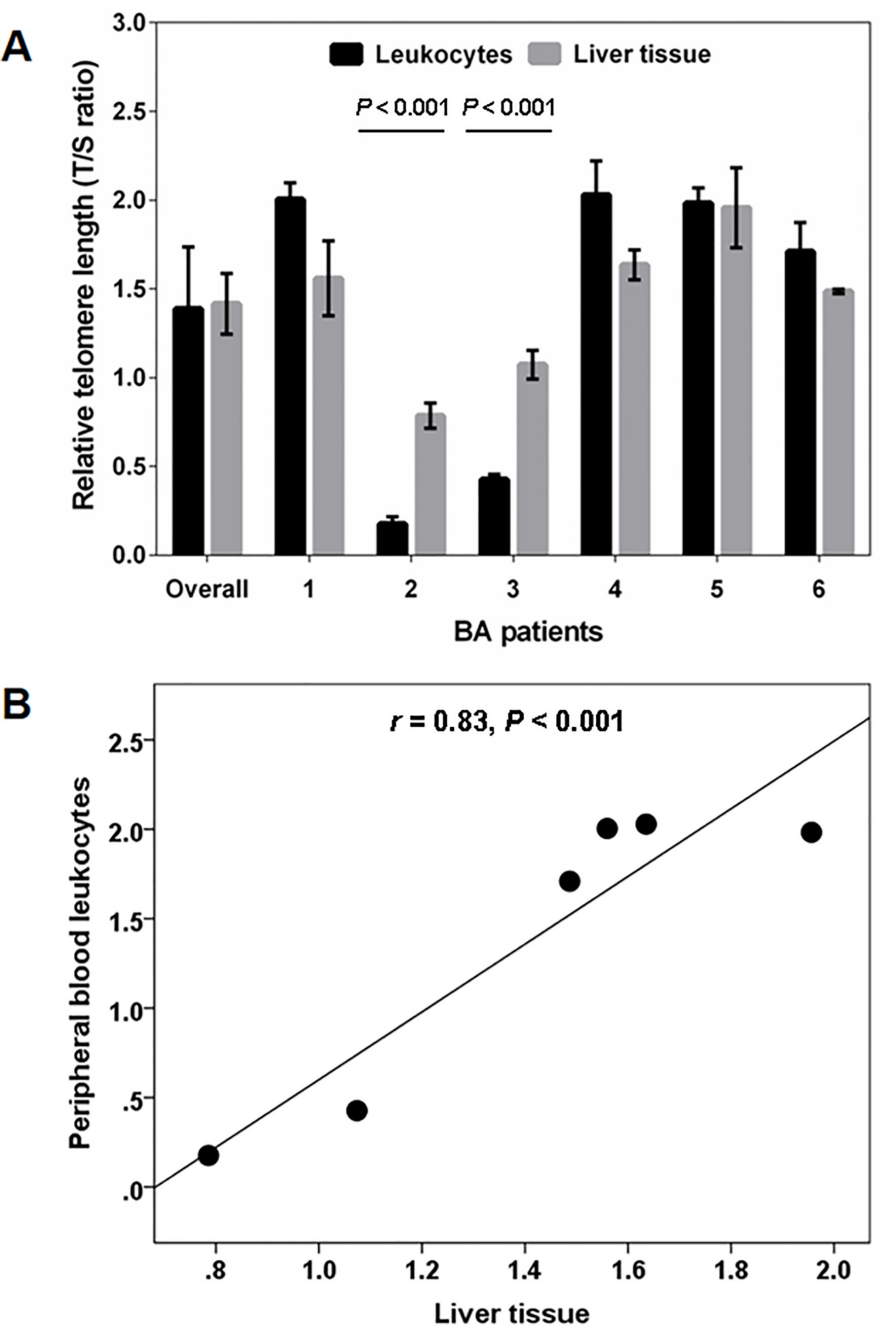
Telomere length distribution between peripheral blood leukocyte and liver tissue in BA patients: (A) Mean levels of relative telomere length for peripheral blood leukocytes and liver tissue in BA patients; (B) Correlation between relative telomere length in peripheral blood leukocytes and liver tissue in BA patients.

Genomic DNA was prepared from matched peripheral blood leukocytes and liver tissue from 6 individuals with BA. Although the RTL was higher in leukocytes compared to liver tissue, the difference was not statistically significant (1.41 ± 0.10 vs 1.36 ± 0.19, respectively), as shown in Fig 5A. Subsequent analysis demonstrated that there was a positive correlation between RTL in peripheral blood leukocytes and RTL in liver tissue (*r* = 0.83, *p* < 0.001; Fig 5B).

## Discussion

In the current study, we examined the relative telomere length of peripheral blood leukocytes in BA patients and investigated the association of telomere length changes with the severity of BA. To the best of our knowledge, this study is the first to demonstrate a dramatically significant decrease in leukocyte RTL in BA patients, as compared to age-matched healthy controls. In addition, advanced BA patients had substantially shorter telomeres than early-stage BA children. We observed that shortened telomere length was associated with a higher risk of liver cirrhosis in BA. Furthermore, RTL was found to be inversely correlated with age and liver stiffness. In contrast, we did not find any relationships between RTL and biochemical parameters such as AST, ALT, ALP, albumin, and total bilirubin in BA children. The ROC curve analysis showed that RTL could be a prognosis indicator for distinguishing BA patients with cirrhosis from non-cirrhosis patients. These findings confirm our hypothesis that a reduction in telomere length is associated with the severity of liver fibrosis in BA and that telomere length may serve as a non-invasive biomarker in determining cirrhosis progression in postoperative BA patients.

The present study has also examined the relationship between leukocyte telomere length and liver telomere length in BA patients. We found that the relative telomere length was not significantly different between leukocytes and liver tissue. Further analysis revealed that RTL in peripheral blood leukocytes was positively correlated with RTL in liver tissue. Our observations are in agreement with a previous study that determined the correlation of leukocyte telomere length with telomere length in liver tissue. Dlouha and coworkers reported a significant direct correlation between leukocyte RTL and liver RTL in human autopsy material [16]. The strong correlation of telomere length between peripheral blood leukocytes and liver tissue suggests that telomere length is relatively similar and that telomeres shorten at approximately similar rates. Our findings support the hypothesis that leucocyte telomere length might have potential as a possible non-invasive biomarker for monitoring the severity and progression of liver cirrhosis in post Kasai BA.

Aberration of the telomere complex might lead to chromosomal and genetic instability, contributing to cellular senescence or apoptosis and increasing the risk of malignancy [6]. Telomere length evaluation is a possibly beneficial biomarker for investigating individual susceptibility for disorders in epidemiological studies, because the balance of processes that abridge and elongate telomeres are largely genetically determined [17, 18]. In addition, a growing body of epidemiological evidence in chronic liver diseases suggested that increased telomere attrition might be closely associated with a genetic risk of liver illness. Here, we report the attrition of telomere length in BA patients. RTL in BA patients was considerably shorter than that in unaffected volunteers, implying that telomere length reduction could be associated with a higher risk of liver cirrhosis in BA. To support this observation, we identified two pairs of female twins, of which only one of the twins was diagnosed with having BA. The twins diagnosed with BA had a shorter telomere length than the healthy twins. Moreover, the leukocyte RTL in the nineteen-year-old twin of set 2 was much lower than that in the nine-year-old twin of set 1. We also observed a significant inverse correlation between leukocyte telomere length and age in patients affected with BA. The explanation for this finding could be due to a progressive decline in leukocyte telomere length with ageing in BA patients.

In accordance with our finding, Kitada et al. investigated telomere length in chronic liver disorders and reported that telomere length was consistently shorter in liver tissue of patients with chronic liver diseases, when compared to control groups [10]. Sanada and colleagues reported hepatocellular telomere length in 20 BA children using quantitative fluorescence *in situ* hybridization that normalized the telomere-centromere ratio in the liver biopsies of the BA group to be significantly smaller than that of the control group [12]. The findings from our study, however, are partially in accordance with the previous research of Invernizzi and colleagues. They found that telomere length in peripheral blood mononuclear cells was not significantly different between patients with primary biliary cirrhosis (PBC) and unaffected volunteers; whereas, an excessive telomere shortening was observed in the advanced stage PBC patients, as compared to healthy controls [19]. The reason for this discrepancy remains unexplained. It may be attributed to a difference in methodology relating to measurement of telomere length between our study and the Invernizzi study.

In humans, decreasing telomere length is correlated with age. Telomere length has been extensively proven to be shorter in patients with age-related disorders than in unaffected volunteers. Its role in mediating age-related disease, however, has not yet been fully elucidated. Our findings also indicate that shortened telomere length in chronological age is significantly different between BA patients and age-matched controls. We further found that telomere shortening showed a trend of inverse association with age in BA patients, suggesting premature cellular ageing in BA children. This is consistent with previous investigations that reported telomere attrition to be negatively correlated with age-related diseases [20, 21].

Chronic liver damage induces regeneration and repair processes in hepatocytes, which leads to elevated cell turnover and ultimately results in excessive telomere shortening. When telomeres become critically shortened, they cause impairment of cell proliferation and senescence. Eventually, hepatocyte growth is arrested and/or senescence assumes a profibrogenic state, either or both trigger the activation of stellate cells by as yet uncertain mechanisms, leading to fibrogenesis in the liver [22]. It is noteworthy that the RTL was considerably shorter in the advanced BA patients with jaundice, when compared with jaundice-free patients, indicating that RTL could be a non-invasive parameter reflecting the severity of biliary atresia.

The mechanism of shortened RTL in leukocytes is not easily addressed. Given the complexity of telomere biology, it merits thorough and complex investigation for clear understanding. Several plausible mechanisms either independently or in combination, may be speculated. In BA, the natural progressive decrease of telomere length with age could be accelerated by telomeric DNA damage due to oxidative stress, chronic inflammation, increased cellular turnover, and/or defects in telomere repair [23]. Telomeric DNA sequences, rich in guanine residues, are likely more susceptible to oxidative stress, particularly by the formation of 8-oxodG. Moreover, these could promote DNA double-strand breaks particularly at telomeric regions resulting in the loss of the distal fragments of telomeric DNA and, thus, telomere shortening with each cell division [24]. Inflammation triggers cellular proliferation and accelerates cell turnover, therefore facilitating telomere attrition due to the end-replication problem. A genetic predisposition must be taken into account. It is conceivable that a variety of these factors may act in concert to generate the phenomenon. The mechanism behind the connection of shortened RTL in leukocytes and liver cirrhosis in BA remains a mystery and requires further study.

This study revealed a significant inverse association between the RTL and liver stiffness in BA patients. Our findings further demonstrated that BA patients with severe fibrosis had increased telomere erosion, compared with BA patients with mild fibrosis, denoting that attrition of telomere length could drive the progression of liver cirrhosis in these patients. These findings are in agreement with results reported by Urabe and collaborators who found that increases in telomere shortening were correlated with the severity of fibrosis in patients diagnosed with human liver diseases [25]. Our findings are also supported by a previous study by Wiemann et al. which demonstrated that telomere shortening in hepatocytes and senescence were associated with fibrotic scarring in human cirrhosis [26]. Thus, the erosion of telomere length is believed to be an indicator of cirrhosis progression and telomere shortening to be an important cause in the pathogenesis of chronic liver injury in BA.

The current research acknowledges certain limitations that should be noted. First, we evaluated telomere length in BA patients, but did not measure the activity of telomerase enzymes. As such, we were not able to determine the effects of telomerase activation and the dynamics of telomere length relating to BA in these results. Second, this study was cross-sectional in its design. Therefore, cause-and-effect associations could not be determined. Prospective longitudinal studies are necessary to investigate the association between telomere shortening and the severity of BA. Third, since this study was carried out with only Thai participants, the results may not be generalizable among other ethnic groups. Fourth, since BA is a sporadic disorder, this study reduced the number of BA subgroups, which diminished the power of the statistics. For this reason, the sample size of BA needs to be increased in order to reach an unequivocal conclusion. Accordingly, the mechanisms behind the connection of shortened RTL in leukocytes and liver cirrhosis in BA remain unknown and need further investigation.

## Conclusion

This study supports the association between short telomere length in leukocytes and higher risk of liver cirrhosis in BA. In addition, RTL in peripheral blood leukocytes was associated with disease severity, showing that BA patients with advanced-stage exhibit excessive telomere shortening. These observations indicate that telomere length measurement might serve as an important predictor of BA patients at high risk of cirrhosis. Prognostic telomere length value as a biomarker for future risk of hepatic impairment in BA needs to be confirmed in a longitudinal study. Further understanding of the pathogenesis of BA will provide new therapeutic approaches to the treatment of this disorder.

## Supporting Information

S1 Table Relative telomere length (T/S ratio) distribution in the study participants. (DOC)
