# Supplementary material for: Telomere Length in Peripheral Blood Leukocytes Is Associated with Severity of Biliary Atresia
Source: PLoS One. 2015 Jul 31;10(7):e0134689. doi: 10.1371/journal.pone.0134689 (PMC4521951; doi:10.1371/journal.pone.0134689)
Supplement: S1 Table — (DOC) [file pone.0134689.s001.doc]

**Supporting information**

**S1 Table.** Relative telomere length (T/S ratio) distribution in the study participants

| **No.** | **Age**  **(years)** | **Ct Telomere** | **Ct Single gene** | **ΔCt**  **(Ct Tel – Ct Single)** | **ΔΔCt**  **(ΔCt Sam - ΔCt Ref 1)** | **2^-(ΔΔCt)** |
| --- | --- | --- | --- | --- | --- | --- |
| **Ref 1** |  | 13.07 | 22.48 | **-9.41** |  |  |
| **Ref 2** |  | 15.10 | 24.45 | -9.35 |  |  |
| **Ref 3** |  | 14.57 | 23.97 | -9.40 |  |  |
| **Negative** |  | Undetermined | Undetermined |  |  |  |
| **Biliary atresia (BA)** | | | | | | |
| **BA 1** | 3.20 | 13.14 | 22.78 | -9.64 | -0.23 | **1.18** |
| **BA 2** | 3.00 | 13.29 | 23.69 | -10.39 | -0.98 | **1.98** |
| **BA 3** | 3.00 | 13.91 | 23.94 | -10.03 | -0.62 | **1.54** |
| **BA 4** | 3.30 | 14.30 | 23.92 | -9.62 | -0.21 | **1.16** |
| **BA 5** | 3.00 | 9.64 | 18.48 | -8.84 | 0.57 | **0.68** |
| **BA 6** | 3.00 | 13.61 | 23.18 | -9.57 | -0.16 | **1.12** |
| **BA 7** | 4.00 | 12.70 | 22.41 | -9.72 | -0.31 | **1.24** |
| **BA 8** | 4.00 | 13.19 | 22.67 | -9.48 | -0.07 | **1.05** |
| **BA 9** | 4.00 | 14.41 | 23.94 | -9.53 | -0.12 | **1.09** |
| **BA 10** | 4.60 | 15.80 | 23.98 | -8.17 | 1.24 | **0.42** |
| **BA 11** | 4.00 | 14.90 | 24.76 | -9.86 | -0.45 | **1.37** |
| **BA 12** | 4.00 | 15.27 | 24.96 | -9.69 | -0.28 | **1.21** |
| **BA 13** | 4.00 | 11.94 | 22.21 | -10.27 | -0.86 | **1.81** |
| **BA 14** | 4.50 | 14.54 | 24.03 | -9.49 | -0.08 | **1.05** |
| **BA 15** | 4.00 | 13.29 | 23.00 | -9.71 | -0.30 | **1.23** |
| **BA 16** | 5.30 | 11.20 | 20.66 | -9.46 | -0.05 | **1.03** |
| **BA 17** | 5.00 | 11.50 | 20.94 | -9.44 | -0.03 | **1.02** |
| **BA 18** | 5.00 | 13.74 | 23.30 | -9.55 | -0.14 | **1.10** |
| **BA 19** | 5.00 | 14.13 | 23.92 | -9.79 | -0.38 | **1.30** |
| **BA 20** | 5.40 | 13.22 | 22.49 | -9.27 | 0.14 | **0.91** |
| **BA 21** | 5.00 | 13.02 | 22.64 | -9.61 | -0.20 | **1.15** |
| **BA 22** | 6.30 | 13.72 | 22.63 | -8.91 | 0.50 | **0.71** |
| **BA 23** | 6.00 | 13.25 | 21.90 | -8.66 | 0.75 | **0.59** |
| **BA 24** | 6.00 | 11.88 | 21.33 | -9.45 | -0.04 | **1.03** |
| **BA 25** | 6.00 | 11.01 | 21.62 | -10.61 | -1.20 | **2.29** |
| **BA 26** | 6.20 | 13.20 | 22.91 | -9.71 | -0.30 | **1.23** |
| **BA 27** | 6.00 | 13.27 | 22.82 | -9.55 | -0.14 | **1.10** |
| **BA 28** | 7.30 | 13.20 | 22.91 | -9.71 | -0.30 | **1.23** |
| **BA 29** | 7.00 | 13.27 | 22.82 | -9.55 | -0.14 | **1.10** |
| **BA 30** | 7.00 | 13.72 | 23.03 | -9.31 | 0.10 | **0.94** |
| **BA 31** | 7.40 | 19.15 | 28.86 | -9.71 | -0.30 | **1.23** |
| **BA 32** | 7.00 | 15.36 | 24.90 | -9.54 | -0.13 | **1.09** |
| **BA 33** | 7.20 | 13.45 | 23.09 | -9.65 | -0.23 | **1.18** |
| **BA 34** | 7.00 | 15.58 | 25.44 | -9.86 | -0.45 | **1.37** |
| **BA 35** | 7.40 | 15.27 | 24.97 | -9.70 | -0.29 | **1.22** |
| **BA 36** | 7.00 | 16.86 | 25.84 | -8.97 | 0.44 | **0.74** |
| **BA 37** | 7.00 | 13.59 | 22.83 | -9.24 | 0.17 | **0.89** |
| **BA 38** | 8.00 | 13.27 | 22.89 | -9.62 | -0.20 | **1.15** |
| **BA 39** | 8.50 | 13.00 | 22.71 | -9.71 | -0.30 | **1.23** |
| **BA 40** | 8.60 | 16.44 | 25.71 | -9.27 | 0.14 | **0.91** |
| **BA 41** | 8.00 | 13.32 | 20.65 | -7.33 | 2.08 | **0.24** |
| **BA 42** | 8.00 | 13.19 | 22.77 | -9.59 | -0.18 | **1.13** |
| **BA 43** | 8.40 | 12.25 | 22.27 | -10.02 | -0.61 | **1.53** |
| **BA 44** | 8.00 | 13.29 | 22.95 | -9.65 | -0.24 | **1.18** |
| **BA 45** | 8.00 | 13.40 | 22.77 | -9.37 | 0.04 | **0.98** |
| **BA 46** | 9.20 | 12.65 | 22.91 | -10.26 | -0.85 | **1.80** |
| **BA 47** | 9.00 | 13.52 | 22.78 | -9.25 | 0.16 | **0.90** |
| **BA 48** | 9.30 | 21.88 | 30.42 | -8.54 | 0.87 | **0.55** |
| **BA 49** | 9.00 | 15.53 | 23.91 | -8.38 | 1.03 | **0.49** |
| **BA 50** | 9.00 | 15.85 | 24.80 | -8.95 | 0.46 | **0.73** |
| **BA 51** | 9.00 | 16.64 | 25.60 | -8.96 | 0.45 | **0.73** |
| **BA 52** | 9.40 | 14.25 | 22.52 | -8.27 | 1.14 | **0.46** |
| **BA 53** | 10.00 | 12.11 | 18.92 | -6.81 | 2.60 | **0.16** |
| **BA 54** | 10.00 | 15.89 | 24.33 | -8.44 | 0.97 | **0.51** |
| **BA 55** | 13.00 | 15.48 | 24.08 | -8.60 | 0.81 | **0.57** |
| **BA 56** | 13.00 | 12.21 | 21.25 | -9.04 | 0.37 | **0.77** |
| **BA 57** | 10.00 | 12.52 | 21.68 | -9.16 | 0.25 | **0.84** |
| **BA 58** | 13.00 | 12.97 | 21.96 | -9.00 | 0.41 | **0.75** |
| **BA 59** | 11.00 | 18.55 | 28.08 | -9.53 | -0.12 | **1.09** |
| **BA 60** | 11.00 | 13.22 | 22.95 | -9.73 | -0.32 | **1.25** |
| **BA 61** | 11.00 | 14.50 | 23.66 | -9.16 | 0.25 | **0.84** |
| **BA 62** | 11.00 | 15.16 | 24.53 | -9.38 | 0.03 | **0.98** |
| **BA 63** | 12.00 | 12.61 | 22.27 | -9.66 | -0.25 | **1.19** |
| **BA 64** | 12.00 | 14.83 | 23.54 | -8.71 | 0.70 | **0.61** |
| **BA 65** | 12.00 | 14.53 | 23.29 | -8.76 | 0.65 | **0.64** |
| **BA 66** | 13.00 | 14.54 | 23.11 | -8.56 | 0.85 | **0.56** |
| **BA 67** | 13.00 | 11.24 | 21.41 | -10.17 | -0.76 | **1.69** |
| **BA 68** | 13.00 | 14.46 | 22.46 | -8.00 | 1.41 | **0.38** |
| **BA 69** | 14.00 | 14.22 | 23.07 | -8.85 | 0.56 | **0.68** |
| **BA 70** | 17.00 | 13.85 | 22.90 | -9.04 | 0.37 | **0.78** |
| **BA 71** | 17.00 | 16.62 | 24.44 | -7.81 | 1.60 | **0.33** |
| **BA 72** | 17.00 | 13.02 | 21.45 | -8.43 | 0.98 | **0.51** |
| **BA 73** | 18.00 | 13.65 | 22.57 | -8.92 | 0.49 | **0.71** |
| **BA 74** | 18.00 | 13.94 | 22.37 | -8.43 | 0.98 | **0.51** |
| **BA 75** | 18.00 | 14.48 | 22.09 | -7.61 | 1.80 | **0.29** |
| **BA 76** | 3.00 | 16.95 | 24.34 | -7.39 | 2.02 | **0.25** |
| **BA 77** | 8.00 | 19.24 | 27.52 | -8.27 | 1.14 | **0.45** |
| **BA 78** | 20.00 | 21.29 | 29.21 | -7.92 | 1.49 | **0.36** |
| **BA 79** | 4.00 | 14.36 | 23.36 | -9.00 | 0.41 | **0.75** |
| **BA 80** | 11.00 | 15.11 | 22.91 | -7.79 | 1.62 | **0.33** |
| **BA 81** | 4.00 | 16.30 | 25.10 | -8.80 | 0.61 | **0.65** |
| **BA 82** | 21.00 | 16.09 | 23.92 | -7.84 | 1.57 | **0.34** |
| **BA 83** | 3.00 | 18.07 | 26.60 | -8.53 | 0.88 | **0.54** |
| **BA 84** | 11.20 | 16.18 | 24.63 | -8.45 | 0.96 | **0.52** |
| **BA 85** | 11.00 | 17.08 | 25.79 | -8.72 | 0.69 | **0.62** |
| **BA 86** | 11.00 | 16.20 | 24.17 | -7.97 | 1.44 | **0.37** |
| **BA 87** | 11.00 | 14.52 | 23.62 | -9.10 | 0.31 | **0.80** |
| **BA 88** | 15.30 | 13.46 | 22.68 | -9.22 | 0.19 | **0.88** |
| **BA 89** | 19.00 | 13.74 | 23.32 | -9.59 | -0.18 | **1.13** |
| **BA 90** | 18.00 | 14.35 | 23.11 | -8.75 | 0.66 | **0.63** |
| **BA 91** | 11.00 | 14.40 | 23.34 | -8.94 | 0.47 | **0.72** |
| **BA 92** | 3.00 | 14.42 | 23.24 | -8.82 | 0.59 | **0.67** |
| **BA 93** | 3.00 | 21.55 | 29.10 | -7.56 | 1.85 | **0.28** |
| **BA 94** | 11.00 | 14.86 | 24.87 | -10.00 | -0.59 | **1.51** |
| **BA 95** | 11.00 | 15.99 | 24.58 | -8.58 | 0.83 | **0.56** |
| **BA 96** | 16.00 | 19.25 | 27.12 | -7.88 | 1.53 | **0.35** |
| **BA 97** | 3.00 | 16.12 | 25.96 | -9.83 | -0.42 | **1.34** |
| **BA 98** | 14.00 | 24.02 | 33.10 | -9.08 | 0.33 | **0.79** |
| **BA 99** | 14.20 | 20.18 | 27.67 | -7.49 | 1.92 | **0.26** |
| **BA 100** | 11.00 | 26.01 | 33.01 | -7.01 | 2.40 | **0.19** |
| **BA 101** | 14.00 | 23.38 | 31.85 | -8.47 | 0.94 | **0.52** |
| **BA 102** | 13.00 | 24.73 | 31.68 | -6.95 | 2.46 | **0.18** |
| **BA 103** | 13.00 | 22.70 | 30.92 | -8.22 | 1.19 | **0.44** |
| **BA 104** | 3.00 | 23.96 | 32.07 | -8.11 | 1.30 | **0.41** |
| **BA 105** | 3.50 | 22.35 | 31.07 | -8.72 | 0.69 | **0.62** |
| **BA 106** | 13.00 | 22.92 | 28.91 | -5.99 | 3.42 | **0.09** |
| **BA 107** | 3.00 | 22.61 | 30.66 | -8.05 | 1.36 | **0.39** |
| **BA 108** | 21.30 | 23.88 | 30.91 | -7.03 | 2.38 | **0.19** |
| **BA 109** | 15.00 | 23.23 | 30.38 | -7.15 | 2.26 | **0.21** |
| **BA 110** | 13.00 | 21.61 | 30.19 | -8.58 | 0.83 | **0.56** |
| **BA 111** | 3.00 | 24.96 | 27.62 | -2.66 | 6.75 | **0.01** |
| **BA 112** | 8.40 | 24.50 | 31.93 | -7.43 | 1.98 | **0.25** |
| **BA 113** | 4.00 | 25.80 | 31.37 | -5.57 | 3.84 | **0.07** |
| **BA 114** | 3.20 | 23.63 | 30.88 | -7.25 | 2.16 | **0.22** |
| **Twin 1 BA** | 9.00 | 15.90 | 24.98 | -9.08 | 0.33 | **0.80** |
| **Twin 1 C** | 9.00 | 15.90 | 24.98 | -9.08 | 0.33 | **0.80** |
| **Twin 2 BA** | 19.00 | 14.56 | 24.83 | -10.27 | -0.86 | **1.82** |
| **Twin 2 C** | 19.00 | 18.30 | 24.76 | -6.47 | 2.95 | **0.13** |
| **Control (C)** | | | | | | |
| **C 1** | 4.00 | 12.10 | 22.93 | -10.83 | -1.42 | **2.68** |
| **C 2** | 4.00 | 11.48 | 21.41 | -9.92 | -0.51 | **1.43** |
| **C 3** | 4.00 | 12.10 | 22.93 | -10.83 | -1.42 | **2.68** |
| **C 4** | 4.00 | 11.48 | 21.41 | -9.92 | -0.51 | **1.43** |
| **C 5** | 4.00 | 11.04 | 22.09 | -11.05 | -1.64 | **3.12** |
| **C 6** | 4.00 | 12.42 | 22.07 | -9.65 | -0.24 | **1.18** |
| **C 7** | 4.00 | 14.43 | 23.70 | -9.27 | 0.14 | **0.91** |
| **C 8** | 5.00 | 15.98 | 25.84 | -9.86 | -0.45 | **1.37** |
| **C 9** | 4.00 | 14.13 | 23.41 | -9.28 | 0.13 | **0.91** |
| **C 10** | 5.00 | 12.10 | 22.93 | -10.83 | -1.42 | **2.68** |
| **C 11** | 5.00 | 11.48 | 21.41 | -9.92 | -0.51 | **1.43** |
| **C 12** | 6.00 | 11.04 | 22.09 | -11.05 | -1.64 | **3.12** |
| **C 13** | 4.00 | 12.42 | 22.07 | -9.65 | -0.24 | **1.18** |
| **C 14** | 3.00 | 14.43 | 23.70 | -9.27 | 0.14 | **0.91** |
| **C 15** | 3.00 | 15.98 | 25.84 | -9.86 | -0.45 | **1.37** |
| **C 16** | 6.00 | 14.13 | 23.41 | -9.28 | 0.13 | **0.91** |
| **C 17** | 3.00 | 15.88 | 25.59 | -9.71 | -0.30 | **1.23** |
| **C 18** | 4.00 | 15.64 | 25.31 | -9.67 | -0.26 | **1.20** |
| **C 19** | 3.00 | 12.91 | 22.62 | -9.71 | -0.30 | **1.23** |
| **C 20** | 6.00 | 12.52 | 21.74 | -9.23 | 0.18 | **0.88** |
| **C 21** | 3.00 | 12.94 | 22.26 | -9.32 | 0.09 | **0.94** |
| **C 22** | 3.00 | 9.95 | 19.75 | -9.80 | -0.39 | **1.31** |
| **C 23** | 3.00 | 14.40 | 24.30 | -9.90 | -0.49 | **1.40** |
| **C 24** | 3.00 | 8.75 | 18.06 | -9.31 | 0.10 | **0.93** |
| **C 25** | 3.00 | 12.86 | 22.78 | -9.93 | -0.52 | **1.43** |
| **C 26** | 3.00 | 13.83 | 23.67 | -9.84 | -0.43 | **1.35** |
| **C 27** | 4.00 | 12.65 | 22.24 | -9.59 | -0.18 | **1.13** |
| **C 28** | 6.00 | 11.95 | 21.92 | -9.96 | -0.55 | **1.47** |
| **C 29** | 3.00 | 8.55 | 18.04 | -9.49 | -0.08 | **1.06** |
| **C 30** | 7.30 | 14.26 | 23.60 | -9.34 | 0.07 | **0.95** |
| **C 31** | 7.00 | 10.92 | 20.06 | -9.14 | 0.27 | **0.83** |
| **C 32** | 7.00 | 12.10 | 21.72 | -9.61 | -0.20 | **1.15** |
| **C 33** | 7.50 | 14.59 | 23.30 | -8.72 | 0.69 | **0.62** |
| **C 34** | 7.00 | 8.23 | 18.08 | -9.85 | -0.44 | **1.36** |
| **C 35** | 7.20 | 13.54 | 23.28 | -9.74 | -0.33 | **1.25** |
| **C 36** | 7.30 | 14.89 | 24.42 | -9.53 | -0.12 | **1.08** |
| **C 37** | 9.00 | 13.64 | 22.63 | -8.99 | 0.42 | **0.75** |
| **C 38** | 9.40 | 13.71 | 23.12 | -9.41 | 0.00 | **1.00** |
| **C 39** | 9.00 | 13.64 | 23.83 | -10.19 | -0.78 | **1.71** |
| **C 40** | 9.20 | 10.57 | 20.05 | -9.48 | -0.07 | **1.05** |
| **C 41** | 9.00 | 9.18 | 19.10 | -9.92 | -0.51 | **1.42** |
| **C 42** | 10.00 | 8.83 | 18.59 | -9.76 | -0.35 | **1.28** |
| **C 43** | 10.60 | 9.09 | 19.09 | -10.00 | -0.59 | **1.51** |
| **C 44** | 11.00 | 9.70 | 18.35 | -8.65 | 0.76 | **0.59** |
| **C 45** | 11.30 | 9.24 | 19.09 | -9.85 | -0.44 | **1.36** |
| **C 46** | 11.00 | 10.22 | 20.03 | -9.81 | -0.40 | **1.32** |
| **C 47** | 11.20 | 12.10 | 22.93 | -10.83 | -1.42 | **2.68** |
| **C 48** | 11.00 | 11.48 | 21.41 | -9.92 | -0.51 | **1.43** |
| **C 49** | 11.60 | 11.04 | 22.09 | -11.05 | -1.64 | **3.12** |
| **C 50** | 11.00 | 12.42 | 22.07 | -9.65 | -0.24 | **1.18** |
| **C 51** | 11.30 | 14.43 | 23.70 | -9.27 | 0.14 | **0.91** |
| **C 52** | 11.00 | 15.98 | 25.84 | -9.86 | -0.45 | **1.37** |
| **C 53** | 11.30 | 14.13 | 23.41 | -9.28 | 0.13 | **0.91** |
| **C 54** | 11.40 | 15.88 | 25.59 | -9.71 | -0.30 | **1.23** |
| **C 55** | 11.00 | 15.64 | 25.31 | -9.67 | -0.26 | **1.20** |
| **C 56** | 11.50 | 12.91 | 22.62 | -9.71 | -0.30 | **1.23** |
| **C 57** | 12.00 | 12.52 | 21.74 | -9.23 | 0.18 | **0.88** |
| **C 58** | 13.20 | 12.94 | 22.26 | -9.32 | 0.09 | **0.94** |
| **C 59** | 13.00 | 9.95 | 19.75 | -9.80 | -0.39 | **1.31** |
| **C 60** | 13.40 | 12.10 | 22.93 | -10.83 | -1.42 | **2.68** |
| **C 61** | 13.00 | 11.48 | 21.41 | -9.92 | -0.51 | **1.43** |
| **C 62** | 13.50 | 11.04 | 22.09 | -11.05 | -1.64 | **3.12** |
| **C 63** | 13.00 | 12.42 | 22.07 | -9.65 | -0.24 | **1.18** |
| **C 64** | 13.00 | 14.43 | 23.70 | -9.27 | 0.14 | **0.91** |
| **C 65** | 13.00 | 15.98 | 25.84 | -9.86 | -0.45 | **1.37** |
| **C 66** | 13.00 | 14.13 | 23.41 | -9.28 | 0.13 | **0.91** |
| **C 67** | 13.20 | 15.88 | 25.59 | -9.71 | -0.30 | **1.23** |
| **C 68** | 14.20 | 15.64 | 25.31 | -9.67 | -0.26 | **1.20** |
| **C 69** | 14.00 | 12.91 | 22.62 | -9.71 | -0.30 | **1.23** |
| **C 70** | 14.50 | 12.52 | 21.74 | -9.23 | 0.18 | **0.88** |
| **C 71** | 14.70 | 12.94 | 22.26 | -9.32 | 0.09 | **0.94** |
| **C 72** | 15.00 | 9.95 | 19.75 | -9.80 | -0.39 | **1.31** |
| **C 73** | 15.80 | 14.40 | 24.30 | -9.90 | -0.49 | **1.40** |
| **C 74** | 16.00 | 8.75 | 18.06 | -9.31 | 0.10 | **0.93** |
| **C 75** | 6.50 | 12.86 | 22.78 | -9.93 | -0.52 | **1.43** |
| **C 76** | 6.00 | 13.83 | 23.67 | -9.84 | -0.43 | **1.35** |
| **C 77** | 7.00 | 12.65 | 22.24 | -9.59 | -0.18 | **1.13** |
| **C 78** | 7.30 | 11.95 | 21.92 | -9.96 | -0.55 | **1.47** |
| **C 79** | 7.00 | 8.55 | 18.04 | -9.49 | -0.08 | **1.06** |
| **C 80** | 8.20 | 14.26 | 23.60 | -9.34 | 0.07 | **0.95** |
| **C 81** | 8.00 | 10.92 | 20.06 | -9.14 | 0.27 | **0.83** |
| **C 82** | 8.00 | 12.10 | 21.72 | -9.61 | -0.20 | **1.15** |
| **C 83** | 8.30 | 14.59 | 23.30 | -8.72 | 0.69 | **0.62** |
| **C 84** | 17.00 | 8.23 | 18.08 | -9.85 | -0.44 | **1.36** |
| **C 85** | 17.00 | 13.54 | 23.28 | -9.74 | -0.33 | **1.25** |
| **C 86** | 18.00 | 14.89 | 24.42 | -9.53 | -0.12 | **1.08** |
| **C 87** | 18.00 | 13.64 | 22.63 | -8.99 | 0.42 | **0.75** |
| **C 88** | 3.00 | 13.71 | 23.12 | -9.41 | 0.00 | **1.00** |
| **C 89** | 3.00 | 13.64 | 23.83 | -10.19 | -0.78 | **1.71** |
| **C 90** | 3.00 | 10.57 | 20.05 | -9.48 | -0.07 | **1.05** |
| **C 91** | 5.00 | 9.18 | 19.10 | -9.92 | -0.51 | **1.42** |
| **C 92** | 5.00 | 12.10 | 22.93 | -10.83 | -1.42 | **2.68** |
| **C 93** | 5.30 | 11.48 | 21.41 | -9.92 | -0.51 | **1.43** |
| **C 94** | 8.00 | 11.04 | 22.09 | -11.05 | -1.64 | **3.12** |
| **C 95** | 8.30 | 12.42 | 22.07 | -9.65 | -0.24 | **1.18** |
| **C 96** | 8.00 | 14.43 | 23.70 | -9.27 | 0.14 | **0.91** |
| **C 97** | 9.00 | 15.98 | 25.84 | -9.86 | -0.45 | **1.37** |
| **C 98** | 9.20 | 14.13 | 23.41 | -9.28 | 0.13 | **0.91** |
| **C 99** | 10.00 | 15.88 | 25.59 | -9.71 | -0.30 | **1.23** |
| **C 100** | 17.00 | 15.64 | 25.31 | -9.67 | -0.26 | **1.20** |
| **C 101** | 18.30 | 12.91 | 22.62 | -9.71 | -0.30 | **1.23** |
| **C 102** | 20.00 | 12.52 | 21.74 | -9.23 | 0.18 | **0.88** |
| **C 103** | 21.50 | 12.94 | 22.26 | -9.32 | 0.09 | **0.94** |
| **C 104** | 21.00 | 9.95 | 19.75 | -9.80 | -0.39 | **1.31** |
| **C 105** | 3.20 | 14.40 | 24.30 | -9.90 | -0.49 | **1.40** |
| **C 106** | 3.00 | 8.75 | 18.06 | -9.31 | 0.10 | **0.93** |
| **C 107** | 8.40 | 12.86 | 22.78 | -9.93 | -0.52 | **1.43** |
| **C 108** | 4.00 | 13.83 | 23.67 | -9.84 | -0.43 | **1.35** |
| **C 109** | 8.50 | 12.65 | 22.24 | -9.59 | -0.18 | **1.13** |
| **C 110** | 8.00 | 11.95 | 21.92 | -9.96 | -0.55 | **1.47** |
| **C 111** | 12.60 | 8.55 | 18.04 | -9.49 | -0.08 | **1.06** |
| **C 112** | 12.00 | 14.26 | 23.60 | -9.34 | 0.07 | **0.95** |
| **C 113** | 18.00 | 10.92 | 20.06 | -9.14 | 0.27 | **0.83** |
| **C 114** | 19.30 | 12.10 | 21.72 | -9.61 | -0.20 | **1.15** |
